# Methods to improve efficacy of orally administered bioactive peptides using bovine colostrum as an exemplar

**DOI:** 10.1371/journal.pone.0253422

**Published:** 2021-06-17

**Authors:** Raymond John Playford, Michael James Weiser, Tania Marchbank

**Affiliations:** 1 Centre for Immunobiology, Blizard Institute, Barts and The London School of Medicine, Queen Mary, University of London, London, United Kingdom; 2 Department of R&D, PanTheryx Inc, Boulder, CO, United States of America; University of Life Sciences in Lublin, POLAND

## Abstract

**Background:**

Oral administration of bioactive peptides has potential clinical advantages, but its applicability is limited due to gastric and pancreatic enzyme proteolysis.

**Objective:**

To examine whether the co-packaging of bovine colostrum (BC), a rich source of IgG, immune and growth factors, with the food additives trehalose (carbohydrate), stearine (fat), casein (protein present in BC) or soy flour (plant based with high protease inhibitory activity) enhances the stability of BC against digestion.

**Design:**

Samples alone and in combination (BC+ 10% wt/wt trehalose, stearine, casein or soy) were exposed to HCl/pepsin, followed by trypsin and chymotrypsin (“CT”). Assessment of proliferation used gastric AGS cells (Alamar blue), IgG function measured bovine IgG anti-*E*.*coli* binding and ELISAs quantified growth factor constituents. In vivo bioassay assessed ability of BC alone or with soy to reduce injury caused by dextran sodium sulphate (DSS, 4% in drinking water, 7 days, test products started 2 days prior to DSS).

**Results:**

Proliferative activity of BC reduced 61% following HCl/pepsin and CT exposure. This was truncated 50% if soy was co-present, and also protected against loss of total IgG, IgG *E*.*coli* binding, TGFβ, lactoferrin and EGF (all P<0.01 vs BC alone). Co-packaging with trehalose was ineffective in preventing digestion whereas casein or stearine provided some intermediate protective effects. Rats given BC alone showed beneficial effects on weight gain, disease activity index, tissue histology and colonic MPO. Soy alone was ineffective. BC+ soy combination showed the greatest benefit with a dose of 7 mg/kg (6.4 BC + 0.6 soy flour) having the same degree of benefit as using 20 mg/kg BC alone.

**Conclusion:**

Soy, and to a lesser extent casein, enhanced the biostability of BC against digestive enzymes. Co-packaging of BC with other food products such as soy flour could result in a decreased dose being required, improving cost-effectiveness and patient compliance.

## Introduction

Use of orally administered growth factors given in isolation or as composites are hampered due to digestion by luminal proteases. Bovine colostrum (BC) is the milk produced during the first few days after birth and has high content of multiple growth factors (such as epidermal growth factor (EGF)), IgG and immune modulators [1]. Orally administered BC has been shown to have benefit for multiple medical conditions such as gut injury caused by nonsteroidal anti-inflammatory drugs, chemotherapy, necrotizing enterocolitis, and inflammatory bowel disease [2–4] and as a supplement to reduce the risk of upper respiratory tract infections in athletes and to aid exercise performance and recovery [5]. Although BC naturally contains serine protease inhibitors, particularly bovine trypsin inhibitor [1], that reduce the rate of proteolysis, most clinical trials have used high doses of BC of approx. 20g/day to overcome this proteolytic issue [e.g., 6]. This results in relatively high cost if used for prolonged periods as a preventative therapy and prevents the use of capsule formulations which are limited to a content of approx. 500 mg/capsule.

For non-peptide therapies, site-specific release formulations are already being used for drugs such as 5-amino salicylic acids to release medication in the colon of patients with ulcerative colitis (UC), increasing local concentrations. Similarly, acid resistant capsules can be used to protect against HCl/pepsin digestion, but also act a barrier so that the active product does not encounter the stomach mucosa. However, none of these approaches are helpful if exposure of wide areas of the gut are preferred, such as reducing the gut damaging effects of NSAIDs on the stomach and on the small and large intestine, where acid suppressants are ineffective and may exacerbate intestinal injury [7] or chemotherapy induced mucositis which also affects the entire GI tract. The clinical challenge is, therefore, to design products that allow the growth factor/immune modulator to interact with the mucosa while reducing its breakdown and loss of biological activity.

To address these problems, we used BC as an exemplar of an orally administered bioactive food supplement. Using in vitro assays, we followed its major constituent structural stability (using immunoassays). In addition, we followed the pro-proliferative activity and IgG-mediated *E*.*coli* binding activity of BC as markers of bio-stability when exposed to digestive enzymes alone and in the presence of additional food industry components that may provide protective benefits. These comprised co-packaging the BC with a carbohydrate (trehalose) or with the protein casein, which is a normal constituent of BC [1] or adding soy flour (as an example of a plant-based food supplement with high protease inhibitory activity). The choice of casein and soya were also based on our previous findings that addition of soya bean trypsin inhibitor (and to a lesser extent casein) was able to preserve bioactivity of recombinant EGF against digestion by human intestinal juice [8]. In addition, we examined the value of coating the BC microgranules with a layer of fat (stearine), a material already being used in the food industry. In a final set of experiments, we progressed the most favorable product to an in vivo DSS-induced rat model of colitis to determine the relative enhancement of activity versus BC alone when used for protection of the distal gut.

## Materials and methods

### BC and other test product samples

Pasteurized BC powder and the combination products were all provided by PanTheryx Inc. (Boulder, CO, USA). BC is collected during the first 24 hours post calving and the subsequent powder, used for all test products contained 64.3% protein (of which 31.8% IgG), 1.8% fat, and the remainder comprising carbohydrates, moisture and ash. Baseline concentrations of growth factors measured for these studies were as follows: EGF 14.99 +/- 1.53 ng/mg powder, TGFβ 26.6 +/- 9.3 pg/mg powder and lactoferrin 3.8 μg/mg powder.

For the BC+ trehalose (TREHA, Hayashibara Co., Okayama, Japan), casein (Excellion EM 9, Friesland Campina, Paramus, New Jersey, USA) and soy flour mixtures, (EASY100, NPI Inc., Grinnell, Iowa, USA) test products were combined while in a liquid form and then spray dried. Briefly, the liquid skim colostrum was mixed thoroughly with trehalose, casein, or enzyme active full-fat raw soy flour. The casein contained 93% protein, 1.5% fat, and 4.5% ash and the soy flour contained 40% protein, 24% fat, 29% carbohydrates, and 8% moisture. The mixture was then spray dried using PanTheryx standard proprietary processes. The resulting powder was collected, bagged, and stored at -20°C.

For the BC+ stearine test product, BC powder was coated in a layer of stearine (derived from hydrogenated soybean oil) using a standard fluid bed unit. The particle size of the powdered colostrum prior to coating was 100–200 μm and was coated with stearine to produce a final product that contained 10% wt/wt stearine. The resulting powder was collected, bagged, and stored at -20°C.

### Cell line

AGS is derived from gastric adenocarcinoma of a 54-year-old female (ATCC, LGC Standards, UK) [9].

### Study 1. Influence of trehelose, stearine, casein and soy flour on in vitro stability

#### Digestion of samples

**Fig 1:**
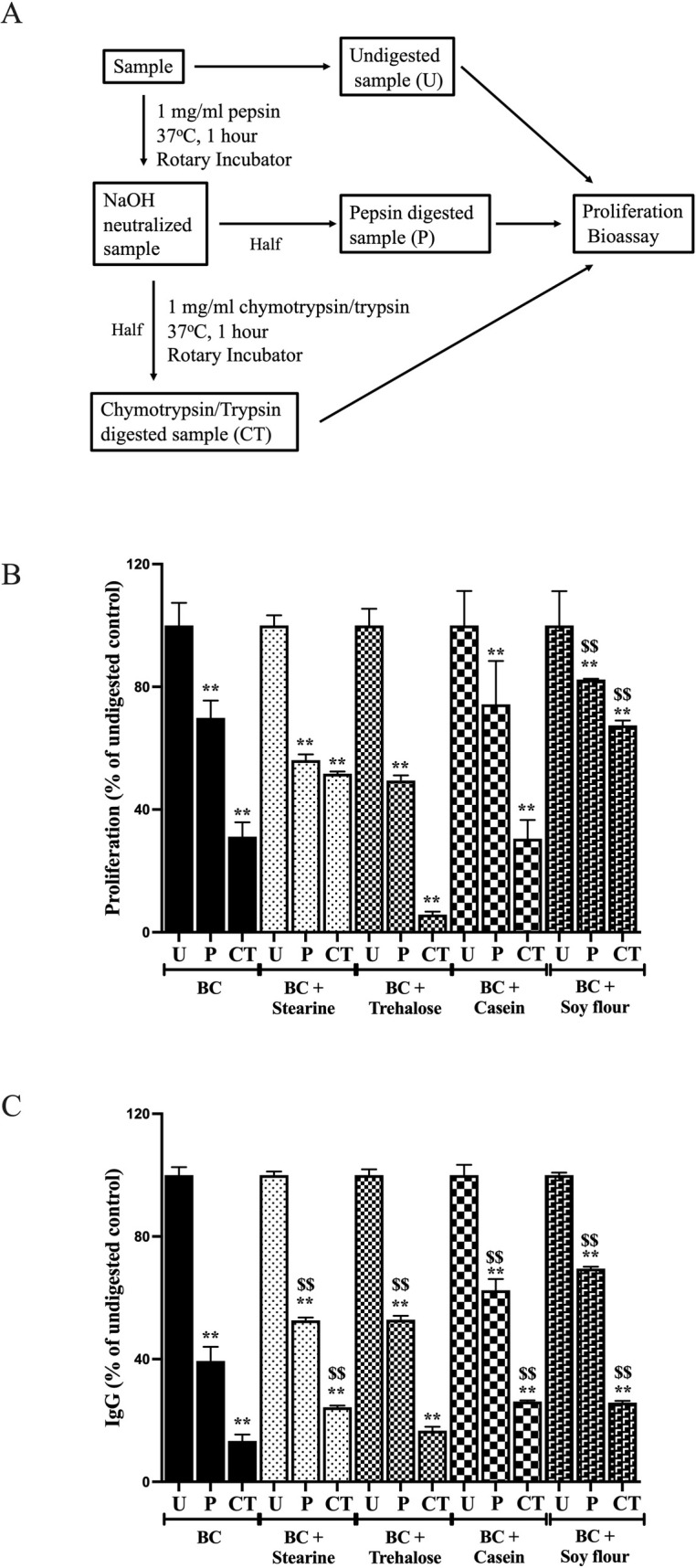
Influence of trehalose, stearine, casein and soy flour on in vitro stability of BC. BC alone or in combination with test products was subjected to digestion protocol (**A**) and analysed for pro-proliferative activity (**B**) (change in A570nm using Alamar blue) and bovine IgG *E*.*coli* binding (**C**). Aliquots of undigested samples (U) were compared against results following HCl/pepsin (P) and chymotrypsin + trypsin (CT) digestion. Results expressed as mean +/- SEM for 4 wells. ** signifies P<0.01 vs undigested sample, $$ signifies P<0.01 vs equivalent digested BC control.

*Protocol*. To reproduce intraluminal exposure of peptides to digestive enzymes within the stomach and small intestine, samples were tested undigested, following exposure to HCl/pepsin alone (1 hour) or HCl/pepsin (1 hour) followed by chymotrypsin and trypsin exposure (1 hour, Fig 1A). Briefly, samples of BC alone or in the combination products (BC + 10% stearine, trehalose, casein or soy flour, 400 mg total weight) were made up in 10 ml of PBS. Samples were then incubated at 37°C without addition of pepsin, chymotrypsin or trypsin (undigested control), or incubated in pepsin (1 mg/ml) pH2 in a rotary incubator at 37°C for 1 hour followed by neutralisation to pH7 using NaHCO_3_. Half of the aliquot was then removed (pepsin digested sample) and the remainder incubated in chymotrypsin and trypsin (“CT”, 1 mg/ml) for 1 hour (CT digested sample). Samples were then analysed using methods described below.

#### Proliferation assays

Cell proliferation assays were performed as previously described, using Alamar blue (Thermofisher Scientific, Hemel Hempstead, UK) [10] as per manufacturer’s instructions. Briefly, cells were seeded at 2000 cells/well, grown in DMEM medium and 10% FCS in 96 well plates overnight. The following day, cells were washed with serum free medium (SFM) and incubated in SFM alone (negative control), or in the presence of undigested or digested samples diluted to identical final concentrations. Samples were all tested at 1 mg powder/ml and results expressed as percentage of baseline (undigested) value.

#### Bovine IgG *E*.*coli* binding

Bovine IgG *E*.*coli* binding was assessed using an ELISA assay. Briefly, 96 well plates were coated with 50 μl Athena strain *Escherichia coli* (MBP-K99) pili (Athena ES, Baltimore, MD) by slowly shaking at room temperature for 2 hours. Plates were washed four times in PBS + 0.05% Tween and blocked by incubation in PBS, 1% BSA, 0.05% Tween 20, shaking slowly for 30 min. Following a wash, samples were added for 1 hour, slowly shaking for 30 minutes at room temperature. Bound bovine IgG was detected using 50 μl rabbit anti-bovine IgG HRP Conjugate (Sigma, A5295-1ml) and TMB substrate (Sigma) and read at absorbance at 450 nm. Preliminary studies determined 1 mg/ml was the optimal for all test samples and results were expressed as percentage of baseline (undigested) values.

#### Growth factor and immunoglobulin levels

Samples from digestion experiments were analysed for levels of IgG and growth factors using commercial ELISA kits according to manufacturer’s instructions. Bovine EGF ELISA (Cusabio, CSB-E17171B) and bovine lactoferrin ELISA (Bethyl laboratories, E11-126) kits were purchased from antibodies-online.com, Aachen, Germany. Bovine TGFβ ELISA (capture ab MAB1835-500, detection ab BAF302 and standard 102-B2-001) and total bovine IgG duoset (DY5930-05) ELISA kits were purchased from R&D systems (Abingdon, UK). Results were expressed as percentage of baseline (undigested) value.

### Study 2. Trypsin inhibitor activity of BC +/- trehalose, stearine, casein and soy flour

Trypsin inhibitor (TI) activity was assessed using a standard Na-benzoyl-DL-arginine-*p*- nitroanilide (BAPNA) trypsin inhibition assay based on the method of Erlander et al. [11] BC was tested at 1 mg/ml and at 0.9 mg/ml (amount of BC present in the combination products). Stearine, trehalose, casein and soy flour were also tested at 0.1 mg/ml (amount present in the BC combination products). Samples were analysed in technical quadruplicates and expressed as mean +/- SEM.

### Study 3. Rat DSS colitis model

#### Ethics

This study was carried out in strict accordance with the recommendations in the Guide for the Care and Use of Laboratory Animals of the National Institutes of Health. All animal experiments were approved by the Local Animals Ethics Committee (Queen Mary’s University of London Animal Welfare Committee) and covered by project (PO13B304A) and personal (IE9346EEF) license under the Home Office Animals Procedures Acts, 1986. Animals were checked daily for any distress and all efforts were made to minimize suffering.

#### Protocol

Male Sprague Dawley rats (225 to 250 g, N = 8 per group; Charles River, UK) were housed in standard cages (five animals per cage) and fed standard laboratory chow (Special Diet Services, Essex, UK) and tap water *ad libitum*. Animals were acclimated for 7 days prior to being placed in the study. A total of 64 animals were used with no mortality throughout the study period.

Methods used were as described by us previously [12]. All rats received a 2 ml gavage daily for 9 days. Negative control group received no DSS and underwent daily gavage with bovine serum albumin (BSA) to determine baseline values. Positive control group received DSS and gavage with BSA. Other groups received DSS along with gavage of BC alone (7 or 20 mg/kg), or BC in combination with 10% soy flour (wt/wt) at a total final combined dose of 7 or 20 mg/kg (i.e., 6.3mg/kg BC+ 0.7 soya flour and 18 mg/kg BC+ 2 mg/kg soy flour). The last two groups received DSS along with soy flour alone (0.7 or 2 mg/kg, i.e., the same amount as used in the BC + soy flour combination).

Colitis was induced by adding 4% (w/v) DSS (molecular mass, 36 to 44 kDa; ICN, Aurora, OH) to the drinking water for 7 days, starting from day 3 of the test product gavage period. Mean DSS and food consumption were noted per cage each day. Rats were weighed daily and visually inspected for signs of distress, diarrhoea, and rectal bleeding. The disease activity index (DAI, based on Cooper et al. [13]) was assessed daily following induction of colitis. The DAI combines the scores of weight loss, stool consistency, and bleeding divided by 3. A cumulative score was then determined over the 7-day DSS treatment period.

At the end of the study, rats were anesthetized by CO_2_ inhalation and euthanized by cervical dislocation, and colonic tissue collected for biochemical and histopathological assessment. Microscopic damage was assessed using the scoring system described by Williams et al. [14]. The total histological colitis score is derived from the sum of the four subscores of i) inflammation severity, ii) inflammation extent, iii) crypt damage, and iv) percentage of involvement. Colonic tissue was also analyzed for myeloperoxidase (MPO) activity (used as a marker of neutrophilic infiltration) as described previously (12).

### Statistics

All results are expressed as mean +/- SEM. Statistics were performed using Graphpad Prism 9 version 9.1.1. Test for normality of data using Shapiro Wilks test showed equal variances between groups. Digestion study data were analysed with a two-way ANOVA, using digestion protocol and test product as factors and using Tukey’s multiple comparison test to compare means between groups. Residual values and F values of the two-way ANOVA are shown in S1 Table. In vivo studies were analysed using one-way ANOVA with treatment as factor. Tukey’s multiple comparison post hoc comparison test was used for multiple comparisons.

## Results

### Study 1. Influence of trehalose, stearine, casein and soy flour on in vitro stability

#### Proliferation

Exposure of BC alone to HCl/pepsin reduced proliferative bioactivity by about 30% with a further 31% reduction following CT exposure. The presence of trehalose, stearine or casein along with the BC did not preserve proliferative bioactivity of BC against HCl/pepsin or CT (Fig 1B). In contrast, co-presence of soy flour preserved the proliferative activity of BC against both HCl /pepsin and CT digestion, improving the final proliferative bioactivity by 52%. Results from 2 way ANoVA.

#### Bovine IgG *E*.*coli* binding

Exposure of BC alone to HCl/pepsin reduced binding by 61% with a further 26% reduction following CT exposure. Presence of stearine did not affect HCl/pepsin digestion but enhanced stability against CT digestion. Trehalose had no beneficial effects against HCl/pepsin or CT. Presence of casein or soya significantly enhanced stability against HCL/pepsin and CT (Fig 1C).

#### IgG levels

**Fig 2:**
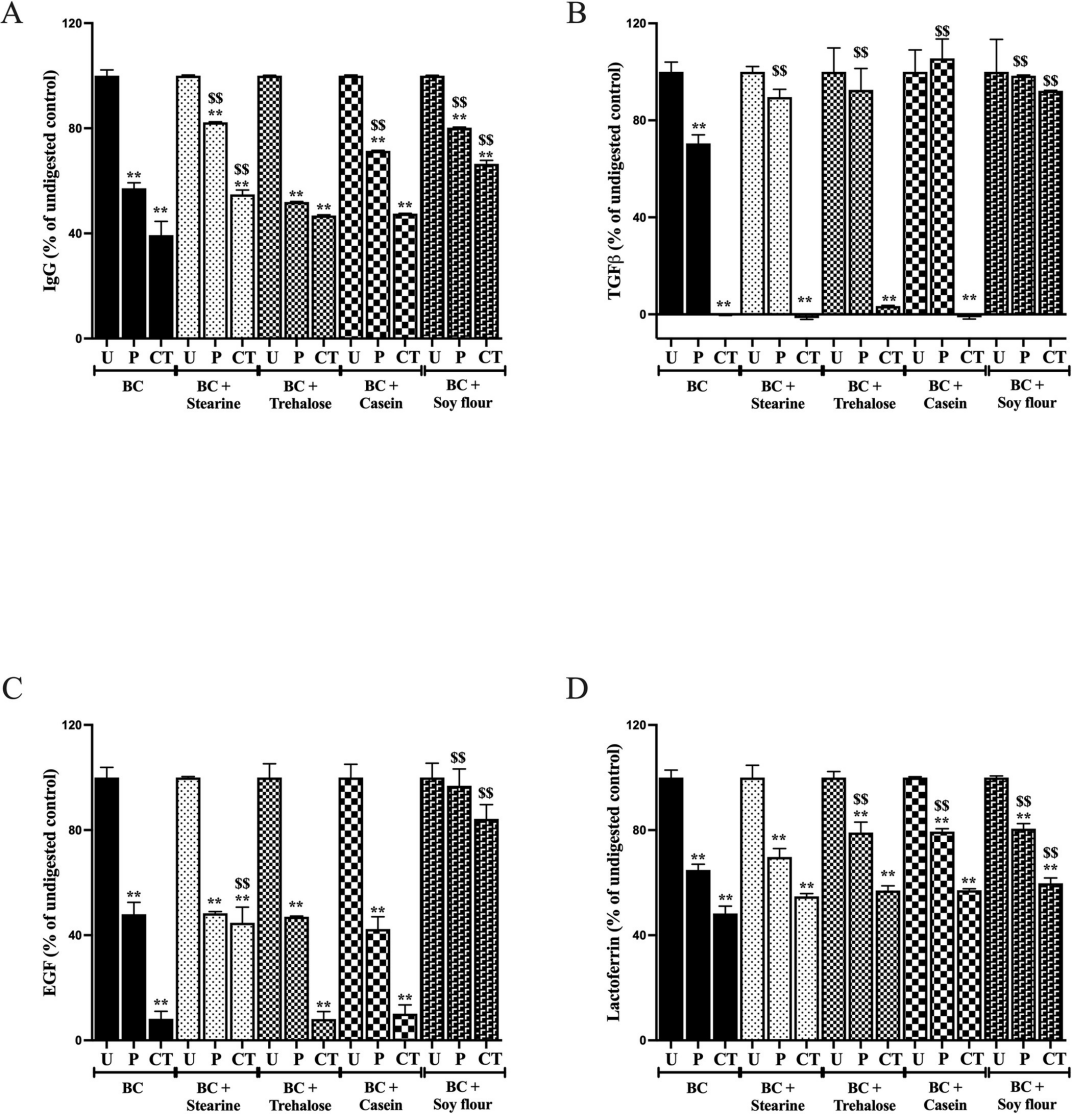
Influence of trehalose, stearine, casein and soy flour on growth factor and IgG immunoreactivity. BC alone, or in combination with test products, was subjected to digestion (see Fig 1A) and analysed by ELISA for effect on total bovine IgG (A), and the growth factors bovine TGFβ (B), bovine EGF (C) and bovine lactoferrin (D). Aliquots of undigested samples (U) were compared against results following HCl/pepsin (P) and chymotrypsin + trypsin (CT) digestion. Results expressed as mean +/- SEM for 4 wells. ** signifies P<0.01 vs undigested sample, $$ signifies P<0.01 vs equivalent digested BC control.

Exposure of BC alone to HCl/pepsin reduced IgG immunoreactivity by 57% and was further reduced by 18% in the presence of CT (Fig 2A). Presence of stearine partially reduced both HCl/pepsin and CT loss of immunoreactivity whereas trehalose had no effect. Casein reduced the loss of immunoreactivity due to HCl/pepsin, but this advantage was lost following CT exposure. Co-presence of soy flour was the most beneficial in preserving immunoreactivity against HCl/pepsin followed by CT, with a 40% improvement over results found when using BC alone.

#### TGFβ

BC exposed to HCl/pepsin alone caused a loss of 30% immunoreactivity with complete loss following CT exposure (Fig 2B). Stearine, trehalose or casein all enhanced TGFβ immunoreactivity stability against HCl/pepsin but were ineffective against CT exposure. In contract, BC in the presence soy flour maintained >90% of its immunoreactivity following HCl/pepsin and CT exposure.

#### EGF

BC alone lost 58% immunoreactivity following HCL/pepsin exposure and a further 39% reduction following CT (Fig 2C). Stearine partially preserved BC against CT exposure whereas trehalose and casein were ineffective. Soy flour was highly effective in preserving activity with EGF immunoreactivity being maintained at 84% of those found in non-digested samples.

#### Lactoferrin

BC alone lost 36% immunoreactivity following HCl/pepsin exposure and a further 16% in response to CT (Fig 2D). Stearine was ineffective in reducing loss of immunoreactivity whereas trehalose and casein truncated the loss of immunoreactivity due to HCl/pepsin exposure. However, neither were effective in preserving immunoreactivity following CT exposure. Co-packaging the BC with soy flour enhanced stability against both HCl/pepsin and CT exposure.

### Study 2. Trypsin inhibitor activity of BC, trehalose, stearine, casein and soy flour alone and in combination

Stearine and trehalose had negligible amounts of TI activity. On a per mg basis soy flour had 12 times TI activity versus casein and 5 times activity of BC. Combination products showed that combining BC with casein or soy flour resulted in enhanced TI activity above that expected from its individual components added together. (Table 1).

**Table 1:** Trypsin inhibitor activity of samples tested alone and in combination.

Sample	μg trypsin inhibited	Theoretical TI from individual constituents	% variation from theoretical
BC (1 mg/ml)	7.55 +/- 0.33	7.55	0
BC (0.9 mg/ml)	6.87 +/- 0.30	6.87	0
stearine (0.1mg/ml)	0.05 +/- 0.05	0.05	0
trehalose (0.1 mg/ml)	0.02 +/- 0.03	0.02	0
casein (0.1 mg/ml)	0.34 +/- 0.09	0.34	0
soy flour (0.1 mg/ml)	4.11 +/- 0.25	4.11	0
BC (0.9mg/ml) + stearine (0.1 mg/ml)	7.52 +/- 0.38	6.87 + 0.05 = 6.92	3.9%
BC (0.9 mg/ml) trehalose (0.1 mg/ml)	7.35 +/- 0.34	6.87 + 0.02 = 6.89	6.2%
BC (0.9 mg/ml) + casein (0.1 mg/ml)	8.66 +/- 0.09	6.87 + 0.34 = 7.21	20.7%
BC (0.9 mg/ml) + soy flour (0.1 mg/ml)	16.03 +/- 0.57	6.87 + 4.11 = 10.98	57.1%

Samples were analysed in quadruplicate with appropriate blanks. Results expressed as mean +/- SEM.

### Study 3. Rat DSS colitis model

#### Body weight changes (Fig 3A)

Rats that did not receive DSS showed an increase of 44 +/- 1.9g over the final 7 days of study. Administration of DSS alone caused the weight gain to be significantly lower (16.2 +/- 2.7g, P< 0.01 compared with no DSS) over the same period. Administration of soy flour alone to DSS treated animals did not enhance weight gain whereas BC caused a dose-dependent increase. Treatment with the BC + soy flour combination showed enhanced results, with the 6.3 mg/kg BC + 0.7 mg/kg soy flour causing the same weight gain as animals given 20 mg/kg BC. The high dose BC + soy four combination (18 mg/kg BC+ 2 mg/kg soy flour) had a weight gain similar to untreated (no DSS) animals.

**Fig 3:**
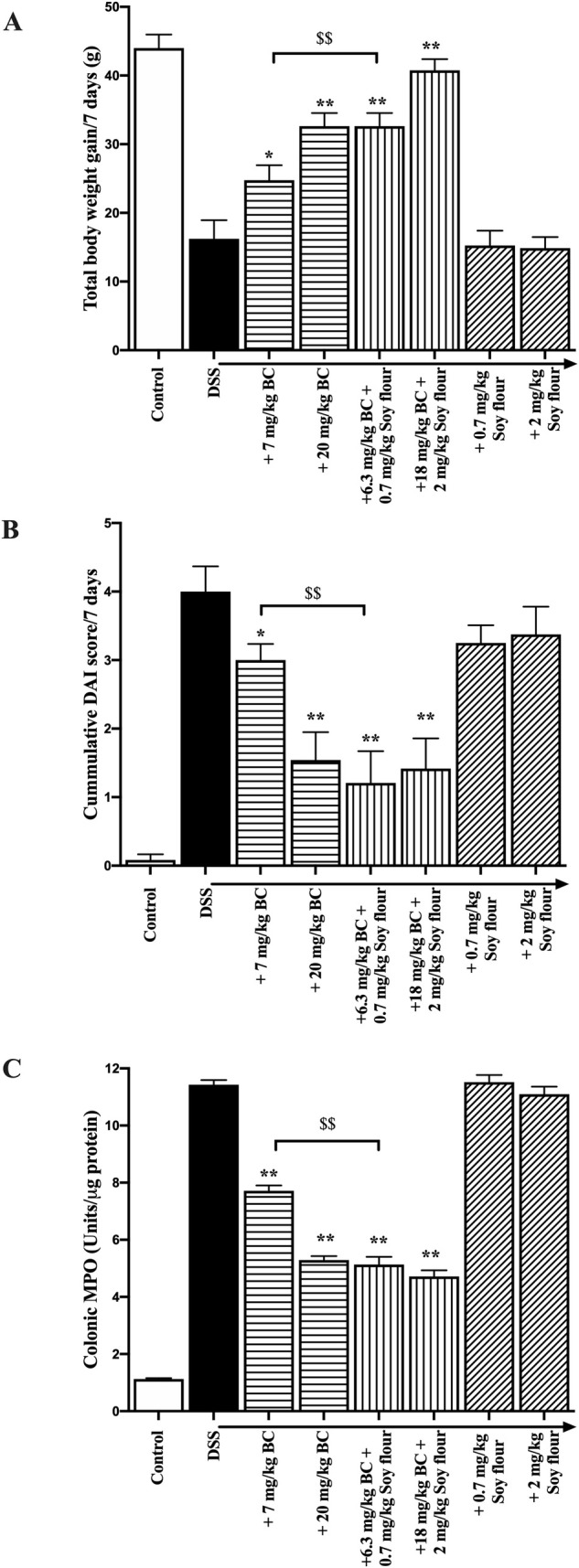
Influence of BC alone, soy flour alone or the combination on DSS-induced colitis in rats. Rats (N = 8/group) received no DSS (normal control) or DSS in drinking water for 7 days. Active treatment groups also received BC alone, BC + soy flour or soy flour alone for 9 days, starting 2 days prior to DSS. Control rats received BSA. (A) Cumulative total body weight changes over 7 days. B). Cumulative DAI score over 7 days [13], (C) Colonic tissue MPO concentrations. Values are means +/- SEM, * or ** signifies P<0.05 or P<0.01, vs DSS alone, respectively. $$ signifies P<0.01 vs DSS + BC alone at 7 mg/kg.

#### DAI score (Fig 3B)

Analyses of DAI scores gave similar results to those seen following weight changes of animals. Soy flour given alone did not affect DAI scores. Dose-dependent beneficial effects were seen using BC alone with the 6.3 mg/kg BC + 0.7 mg/kg soy flour combination product reducing DAI score to a similar extent to using high dose (20mg/kg) BC alone.

#### Colonic MPO levels (Fig 3C)

Results from MPO analyses were in keeping with body weight changes and DAI scores. Administration of DSS alone caused a 11-fold increase in colonic MPO. Administration of soy flour to DSS treated animals alone did not improve MPO levels. In contrast, both doses of BC alone and BC + soy flour truncated the rise in MPO concentrations caused by DSS (P *<* 0.01), with the 6.3 mg/kg BC + 0.7 mg/kg soy flour combination product reducing MPO to a similar extent to using high dose (20mg/kg) BC alone.

#### Histological assessment

**Fig 4:**
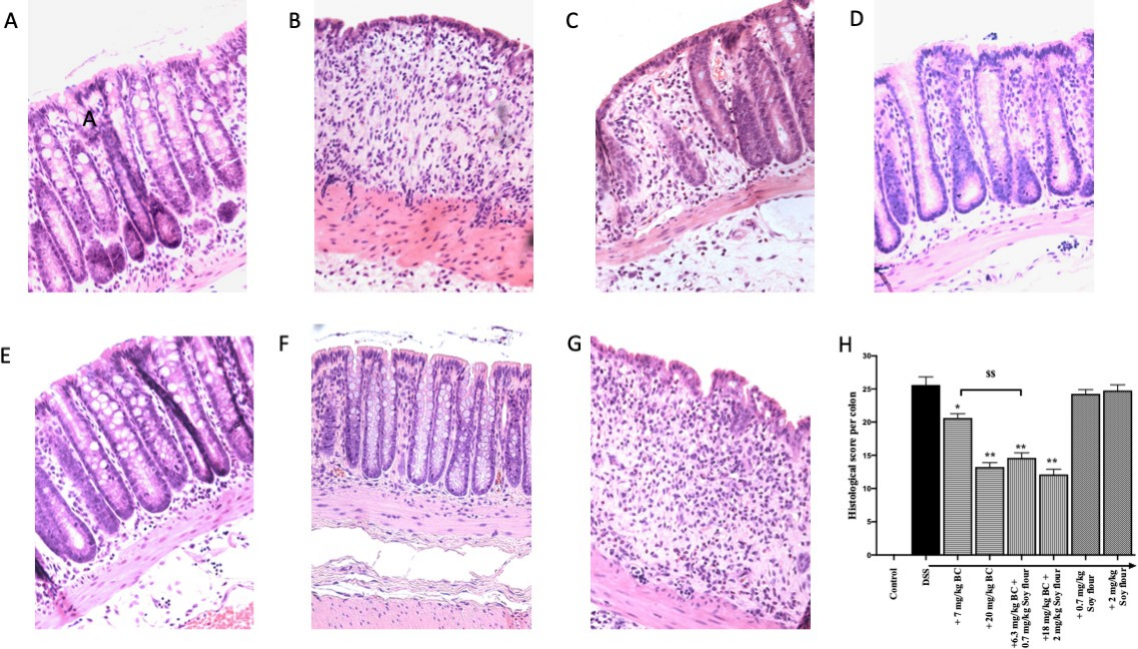
Influence of BC alone, soy flour alone or the combination on DSS-induced histological colonic injury in rats. Same rats as Fig 4. (A) Normal (no DSS), showing normal crypt structures (B) DSS alone, showing complete loss of crypt architecture and increased inflammatory infiltrate (C) DSS + BC (7 mg/kg), (D) DSS + BC (20 mg/kg), (E) DSS + BC (6.3 mg/kg) + soy flour (0.7 mg/kg), (F) DSS + BC (18 mg/kg) + soy flour (2 mg/kg), (G) DSS + soy flour alone (2 mg/kg), (H) Histological scores of rat colons using method described in [14].

Morphology showed that compared with normal (no DSS) controls (Fig 4A), administration of DSS caused almost complete loss of normal crypt structure combined with major infiltration of inflammatory cells (Fig 4B). Treatment with soy flour alone (Fig 4G) did not influence the damaging effect of DSS. Improvement was seen in animals that received 7 mg/kg BC alone, with the inflammatory infiltrate being less marked and crypt structure partially maintained (Fig 4C). Animals that received 20 mg/kg of BC alone (Fig 4D) or either dose of BC + Soy flour (Fig 4E and 4F) showed major improvement with minimal inflammatory infiltrate, and maintenance of crypt structures. Histological scoring (Fig 4H) was consistent with the morphological results.

## Discussion

Using a combination of in vitro and in vivo models, we examined the susceptibility of BC to digestion from gastric and pancreatic enzymes and the beneficial effects of packaging BC with a variety of food materials to maintain bioactivity.

Use of gastrointestinal cell lines to determine the relative proliferative bioactivity of peptides/proteins and the effect of partial proteolysis on bioactivity has been used by ours and other groups previously. For example, we showed EGF(1–51) and EGF(1–52) are as biologically active as full length EGF(1–53) [15] whereas EGF(1–49), which is produced by HCl/pepsin digestion, loses approximately 75% of its proliferative and reparative activity [16]. The gastric cell line AGS was chosen as it is of human origin, correlates well with results testing BC using intestinal or colonic cell lines [17] and remains viable in the presence of pancreatic proteolytic enzymes.

We progressed our studies to examine the effect of supplementing BC with food industry products to potentially enhance preservation against digestion. This consisted of co-packaging the BC in a spray dried formulation along with the sugar (trehalose), the milk-based casein or the plant-based soy flour. The final combination product that was tested comprised BC which was initially spray dried as normal and then externally spray coated with the fat stearine. Each combination product was assessed at 10% wt/wt of BC content to maintain consistency. Trehalose is a naturally occurring food ingredient found in mushrooms, sunflower seeds and yeast. It is a nonreducing sugar formed from two glucose units joined by a 1–1 alpha bond, giving it the name α-D-glucopyranosyl-(1→1)-α-D-glucopyranoside. The bonding makes trehalose very resistant to acid hydrolysis and it has a wide range of applications in foods, beverages, pharmaceutical and cosmetics industries [18]. However, trehalose encapsulation proved largely ineffective in preserving bioactivity or biostability.

Stearine (also known as tristearin, or glyceryl tristearate), is an odourless triglyceride derived from three units of stearic acid. It is commonly used in baked goods and confectionary and for production of soft margarines and in infant fat formulas [19]. Combining BC with stearine did not enhance TI activity, was not effective in maintaining proliferative activity but did enhance stability in some of the other assays.

We examined the effect of casein supplementation on BC stability as it is naturally present in BC and is used commercially as a relatively cheap protein supplement, particularly for athletes and body builders [20]. BC + casein enhanced TI activity compared to the sum of the individual components. Its co-presence enhanced stability of many of the components of BC against destruction by HCl/pepsin. Following CT exposure, casein partially preserved the IgG biostability (IgG E Coli binding) of BC but not its pro-proliferative activity.

Soy flour is used by food industry as a cheap protein source [21]. Soy flour proved efficacious in preserving biostability and bioactivity for all the tested parameters and was therefore selected for further investigation using an in vivo model of gut injury. The beneficial effect of soy flour is likely to be through acting as a competitive substrate for the proteases due to its TI content. As expected, BC contained lower amounts of total TI activity than soy flour but was still approx. twice that found in casein. TI activity of BC is contributed to by bovine trypsin inhibitor which is present at about 100 times higher concentration in BC than mature milk [22]. As seen with BC+ casein, an unexpected finding was the synergistic TI activity if BC and soy flour were added together. The molecular reason behind this is unclear as the TI sites of both BC and Kunitz soybean trypsin inhibitor (SBTI) have a single active site [23, 24]. This synergy may, nevertheless, contribute to the beneficial effects seen in the in vivo and in vitro models.

Our previous studies demonstrated a six-fold difference in proliferative bioactivity of commercially available BC, despite the products having similar total protein and IgG levels [17]. This led us to suggest that some form of bioassay, such as a proliferation assay, should occur to reassure customers of consistency of product, particularly if being used for medical purposes. The current studies suggest a functional bioassay should also be used for IgG function as for example, the preservative effect of adding soya to BC appeared twice as effective if measuring immunoreactive stability using an ELISA, as opposed to a true bioassay (IgG functional *E*.*coli* binding) which probably has more physiological relevance.

We chose an in vivo model of UC to study the potential beneficial effect of soy flour supplementation of BC. UC is a chronic relapsing disease, where powerful immunosuppressants are often used but may result in serious side effects such as immune suppression and increased risk of infections. Methods to induce colitis in animal models include administrating a noxious compound either orally or rectally (so-called *inducible colitis* models) or studying rats or mice that have been genetically modified to cause *spontaneous colitis*, due to an excessive immune response. We used oral administration of DSS as we have previous experience of using this model to test peptide growth factors [12, 25] and it allows the researcher to determine the temporal relationship between test product administration and the induction of colitis. Induction of DSS colitis is mediated through the animal’s innate immune system, causing alteration in both Th1 and Th2 cytokine profiles, although the Th1 response predominates [26]. To allow a reasonable number of animals and groups to be compared within a single experiment, two doses of BC, soy flour and BC + soy flour were tested alongside positive and negative controls. Results from the in vivo study were consistent across all the measured parameters of DSS-induced injury. Soy flour alone did not affect markers of inflammation or injury whereas BC caused a dose-dependent reduction in all markers. The combination of BC with soy flour provided an approximate 3-fold enhancement in protective activity.

BC contains multiple bioactive molecules that can stimulate repair e.g., EGF, TGFβ, IGF-I [1], with the EGF receptor (EGFR) and TGFβ-receptor pathways having both been shown to be relevant in mediating reparative effects of BC [17]. Although many of the growth factor receptors on gut cells, such as the EGFR, are basolaterally distributed [27] they become accessible to luminal growth factors at sites of injury [28]. BC also contains factors that influence antioxidant and immune function e.g., interleukin (IL) 1β, IL-6, IL-10, TNFα and lysozyme [1]. It is, therefore, probable that multiple molecules were responsible for the beneficial effects. Further studies would be required to determine the contribution of individual components to the beneficial effects seen, although the in vivo situation is more complex with tissues exposed simultaneously to multiple factors that can result in synergistic responses. For example, synergistic proliferative responses were found when EGF and bovine lactoferrin (both components of BC) were added together to rat intestinal IEC-18 cells [29]. For a detailed review of bioactive constituents of BC see ref [1]. Similarly, isolated SBTI could be used in place of soy flour, however, purified SBTI is markedly more expensive and potentially moves the combination to a biological/pharmaceutical product rather than a food supplement or medical food.

Although caution must be shown in extrapolating from the animal model to the human situation, our studies have potential clinical relevance; BC is currently used by the public for general GI and immune support and shows promise for treating or preventing multiple GI conditions [2–5]. Most clinical trials have used doses of BC of approx. 10-20g/day to elicit clinical effects [e.g., 6], resulting in relatively high cost if used for prolonged periods. Such large amounts are also impractical to deliver by capsule which can only accommodate approx. 500 mg/capsule. It is probably for these cost and compliance reasons why most food supplement products recommend much lower doses 500mg-1g/day (i.e., 1 or 2 capsules), although the evidence base for clinical or physiological relevance at these lower doses is more limited. Effective co-packaging of BC with other food products such as soy flour to preserve bioactivity could result in a decreased dose being required, improving cost-effectiveness and patient compliance.

## Supporting information

S1 Table Two-way ANOVA result for study 1. Influence of trehalose, stearine, casein and soy flour on in vitro stability of BC following digestion by HCl/pepsin alone, or HCl/pepsin followed by trypsin/chymotrypsin digestion.
(DOCX)

S1 Checklist The ARRIVE guidelines 2.0: Author checklist. (PDF)

## Abbreviations

BC: bovine colostrum
DSS: dextran sodium sulphate
EGF: Epidermal growth factor
EGFR: epidermal growth factor receptor
Ig: immunoglobulin
IGF: insulin-like growth factor
MDGF: milk derived growth factor
MFG-E8: Milk fat globule-EGF factor 8
NSAID: Nonsteroidal anti-inflammatory drugs
PDGF: platelet derived growth factor
SBTI: soybean trypsin inhibitor
SFM: serum free medium
TI: trypsin inhibitor
TGF: transforming growth factor
VEGF: vascular endothelial growth factor
