# Supplementary material for: Methods to improve efficacy of orally administered bioactive peptides using bovine colostrum as an exemplar
Source: PLoS One. 2021 Jun 17;16(6):e0253422. doi: 10.1371/journal.pone.0253422 (PMC8211160; doi:10.1371/journal.pone.0253422)
Supplement: S1 Table — Influence of trehalose, stearine, casein and soy flour on in vitro stability of BC following digestion by HCl/pepsin alone, or HCl/pepsin followed by trypsin/chymotrypsin digestion. (DOCX) [file pone.0253422.s001.docx]

| Parameter tested | Figure panel | Effect of test product | Effect of digestion protocol | Effect of test product X digestion interaction | Residual |
| --- | --- | --- | --- | --- | --- |
| Proliferation | 1B | F_(4, 45)_ = 8.702  P<0.001 | F_(2, 45)_ = 114.1  P<0.0001 | F_(8, 45)_ = 4.107  P=0.001 | 172.4 |
| Bovine IgG *E.coli* binding | 1C | F_(4, 45)_ = 20.56  P<0.0001 | F_(2, 45)_ = 1722  P<0.0001 | F_(8, 45)_) = 7.894  P<0.0001 | 18.11 |
| IgG levels | 2A | F_(4, 45)_ = 61.02  P<0.0001 | F_(2, 45)_ = 1123  P<0.0001 | F_(8, 45)_ = 22.24  P<0.0001 | 10.97 |
| TGFβ: | 2B | F_(4, 45)_ = 20.27  P<0.0001 | F_(2, 45)_ = 277.1  P<0.0001 | F_(8, 45)_ = 15.78  P<0.0001 | 144.6 |
| EGF | 2C | F_(4, 45)_ = 55.13  P<0.0001 | F_(2, 45)_ = 333.1  P<0.001 | F_(8, 45)_ = 17.47  P<0.0001 | 72.9 |
| Lactoferrin | 2D | F(_(4, 45)_ = 7.12  P=0.0002 | F_(2, 45)_ = 427.4  P<0.001 | F_(8, 45)_ = 2.261  P=0.04 | 23.41 |

**S1 Table:** Two-way ANOVA results for Study 1. Influence of trehalose, stearine, casein and soy flour on in vitro stability of bovine colostrum following exposure to HCl/pepsin and trypsin and chymotrypsin.
